# Predictive value of thyroid autoantibodies for coronary heart disease severity in individuals with normal thyroid function based on machine learning and SHAP interpretation

**DOI:** 10.3389/fimmu.2026.1803188

**Published:** 2026-05-12

**Authors:** Nana Liang, Huiru Ma, Xingyue Wang, Jie Meng, Zixuan Ru, Na Lv, Kerou Li, Hong Qiao

**Affiliations:** The Second Affiliated Hospital of Harbin Medical University, Harbin, China

**Keywords:** cardiovascular disease, machine learning, SHAP, TgAb, TPOAb

## Abstract

**Objective:**

Assessing the severity of coronary heart disease (CHD) is critical for clinical decision-making. Thyroid autoantibodies are associated with cardiovascular disease, but their ability to predict CHD severity remains unclear. This study aimed to systematically elucidate the predictive value of thyroid autoantibodies for CHD severity using machine learning methods.

**Methods:**

This retrospective study included 942 patients hospitalized in the cardiovascular department between January 2024 and June 2025, comprising 590 patients with severe lesions and 352 with nonsevere lesions. Traditional statistical analysis employed correlation analysis and multivariate logistic regression. Eight machine learning models were subsequently constructed and compared: logistic regression (LR), support vector machine (SVM), K-nearest neighbors (KNN), adaptive boosting (AdaBoost), multilayer perceptron (MLP), random forest (RF), gradient boosting (GB), and XGBoost (XGB). The optimal model was determined using Shapley additive propagation (SHAP), and the robustness of the core findings was validated through hierarchical analysis and subgroup feature importance comparisons.

**Results:**

Multivariate logistic regression revealed that log anti-TPO was an independent risk factor for severe coronary lesions (OR = 2.19; 95% CI: 1.87–2.57; P<0.001), whereas log anti-Tg shows a negative correlation after adjustment (OR = 0.72; 95% CI: 0.61–0.85; P<0.001). However, given the significant multicollinearity between the two variables (r = 0.55), this negative correlation strongly suggests that it is a statistical artifact. The gradient boosting tree performed best (AUROC:0.855). SHAP analysis consistently confirmed three key predictive features: log anti-TPO, log anti-Tg, and glycated hemoglobin (HbA1c). SHAP dependency plots further revealed a distinct threshold effect for log anti-TPO, while elevated log anti-Tg was associated with reduced risk, However, this negative association is likely a statistical artifact rather than an independent protective effect. Stratified analysis and sex-specific feature importance assessments confirmed that log anti-TPO demonstrated a highly robust and significant predictive value across all the subgroups, ranking as the primary predictor in both the male and female cohorts.

**Conclusion:**

The presence of thyroid autoantibodies represents an independent key predictor of CHD severity. Thyroid peroxidase antibodies (TPO-Ab) serve as a strong risk marker, The gradient boosting tree model demonstrated optimal predictive performance when these biomarkers were integrated.

## 1 Introduction

Coronary heart disease (CHD) is a chronic cardiovascular condition caused by atherosclerosis of the coronary arteries. Owing to population aging, urbanization, obesity, and unhealthy lifestyles, the prevalence and mortality rates of CHD are on the rise. Statistics indicate that the global CHD patient population has reached 197.2 million, posing a severe public health challenge (1–5). The severity of coronary artery disease varies among patients and requires the assessment of clinical symptoms and auxiliary examinations. Coronary angiography (CAG), which serves as the gold standard for diagnosing coronary heart disease, can be used to precisely detect the number and degree of arterial stenoses (6). However, owing to its high cost and invasive nature, some patients are unable to undergo coronary angiography for screening or long-term monitoring. Over the past decade, significant advances have been made in treating and preventing the progression of coronary heart disease by targeting modifiable risk factors, including diabetes, insulin resistance, obesity, hyperlipidemia, and metabolic syndrome. Nevertheless, even after optimal management of traditional risk factors, numerous additional risk factors remain that may influence the progression of coronary heart disease (7–11). Therefore, exploring novel biomarkers is important for improving disease progression.

Thyroid autoimmune disease is a classic, organ-specific autoimmune disorder characterized by the production of thyroid autoantibodies and lymphocytic infiltration of the thyroid gland (12). Serum thyroid autoantibody titers that are persistently at or greater than the upper limit of normal constitute the minimum diagnostic criteria for thyroid autoimmunity (7). Reports have indicated that the prevalence rates of TPO-Ab and Tg-Ab in the general Chinese population are as high as 11.5% and 12.6%, respectively (8). Positive serum thyroid autoantibodies may lead to thyroid dysfunction. Abnormal thyroid function is a risk factor for cardiovascular disease (11). Among individuals with thyroid dysfunction, the relationship between thyroid autoantibodies and cardiovascular disease has been extensively studied; however, most studies have failed to establish a significant association between the two (13–20). All of the above studies included populations with thyroid dysfunction, leading to conclusions influenced by the confounding factor of thyroid hormone levels. Notably, in recent years, research has focused on the impact of thyroid autoantibodies on coronary heart disease in individuals with normal thyroid function. For example, A meta-analysis encompassing 14 cohorts demonstrated that TPO-Ab did not increase the risk of coronary heart disease or stroke in either the general population (20). It can be concluded that, in individuals with normal thyroid function, thyroid autoantibodies themselves are not an independent trigger for cardiovascular events. On the contrary. Cross-sectional studies have reported a positive correlation between TPO-Ab titers within the normal range and atherosclerosis (21). However, most studies have focused on the presence or absence of coronary heart disease while neglecting to assess its severity. Coronary heart disease is a highly heterogeneous condition, with significant variations in clinical prognosis and treatment strategies. While thyroid autoantibodies may not initiate the development of atherosclerosis, once atherosclerosis has established itself, the autoimmune state may modulate the severity and progression of the disease through mechanisms such as influencing plaque stability, the intensity of the inflammatory response, and the reparative capacity of the vascular endothelium. Based on this, the aim of this study is to investigate whether TPO-Ab status has independent predictive value for the severity of coronary artery disease in individuals with normal thyroid function who have been diagnosed with coronary heart disease.

In recent years, artificial intelligence (AI) has demonstrated significant advantages in disease prediction. Machine learning, encompassing deep learning (DL) and traditional machine learning methods, is the most widely adopted technology (22). Traditional machine learning has emerged as a powerful computational method for data analysis and has gained widespread acceptance in the medical field as an effective tool for predicting disease risk (23–25). Previous studies have employed linear or logistic regression models, which have limitations in handling complex nonlinear relationships and interactions among variables, potentially leading to inaccurate results. In contrast, machine learning methods effectively capture nonlinear relationships within data using sophisticated internal algorithms, offering unique advantages when processing high-dimensional data. These approaches are being increasingly used to complement traditional statistical techniques (22). An increasing number of researchers are employing machine learning to explore the complex associations between thyroid disorders and coronary heart disease. However, evidence regarding whether thyroid autoantibodies can independently predict the severity of coronary heart disease remains limited and inconclusive. Therefore, this study aimed to use machine learning methods to evaluate the predictive value of thyroid autoantibodies for determining coronary heart disease severity in individuals with normal thyroid function.

## 2 Materials and methods

### 2.1 Study design and patients

This study included 942 patients who underwent their first coronary angiography during hospitalization in the Department of Cardiology at the Second Affiliated Hospital of Harbin Medical University between January 2024 and June 2025. The inclusion criteria were as follows: (1) age ≥18 years; (2) normal thyroid function; (3) confirmed coronary artery disease; and (4) availability of complete medical records, laboratory test results, and other necessary medical documentation. The exclusion criteria were as follows: (1) a history of thyroid dysfunction; (2) current use of medications affecting thyroid function (e.g., levothyroxine, antithyroid drugs, amiodarone); (3) a history of coronary revascularization surgery; (4) severe hepatic or renal insufficiency; (5) acute or chronic infections; and (6) a history of heart failure, arrhythmia, cardiomyopathy, myocardial infarction, valvular heart disease, pericardial disease, or infective endocarditis.

### 2.2 Ethics statement

All patient data collection and analysis in this retrospective study were conducted anonymously to protect patient privacy. Furthermore, this human research complied with the provisions of the Declaration of Helsinki and was approved by the Medical Ethics Committee of the Second Affiliated Hospital of Harbin Medical University (Approval Number: YJSDW2025-156).

### 2.3 Disease definition

All the patients in this study underwent coronary angiography via the radial or femoral artery—the gold standard for diagnosing coronary artery disease and assessing stenosis severity—with results jointly determined by two cardiologists. On the basis of coronary angiography findings, the patients were categorized into two groups: severe disease (non-left main lesions ≥70% diameter stenosis or left main lesions ≥50%) and non-severe disease (non-left main lesions <70% diameter stenosis and left main lesions <50%) (26). The diagnostic criteria for diabetes mellitus included typical clinical manifestations (excessive thirst, frequent urination, increased appetite, weight loss) plus any one of the following: a venous plasma glucose concentration ≥11.1 mmol/L (at any time point), a fasting plasma glucose concentration ≥7.0 mmol/L, a 2-hour value ≥11.1 mmol/L on an oral glucose tolerance test (OGTT), a glycated hemoglobin (HbA1c) concentration ≥6.5%, or a prior diagnosis of diabetes (27). Hypertension was defined as an average systolic blood pressure (SBP) ≥140 mmHg and an average diastolic blood pressure (DBP) ≥90 mmHg, current treatment with antihypertensive medication, or a diagnosis confirmed by an internist (28). Smokers were defined as individuals with a lifetime history of continuous or cumulative smoking of ≥6 months. The alcohol consumption history included drinking for more than 5 years with a daily intake of ≥40 grams for men and ≥20 grams for women or having engaged in binge drinking within the past 14 days.

### 2.4 Data collection

General patient information and clinical data, including sex, age, height, weight, blood pressure, smoking history, alcohol consumption history, hypertension status, history of diabetes, triglyceride (TG), total cholesterol (TC), low-density lipoprotein (LDL), high-density lipoprotein (HDL), glucose (Glu), HbA1c, B-type natriuretic peptide (BNP), cardiac troponin (CTn), free triiodothyronine (FT3), free thyroxine (FT4), thyroid-stimulating hormone (TSH), TgAb, TPO-Ab, electrocardiogram (ECG), echocardiogram (UCG), and coronary angiography (CAG), were collected. Body mass index (BMI) was calculated using the following formula: weight (kg)/height² (m²). The normal reference ranges for FT3, FT4, TSH, TPO-Ab, and TgAb are 2.43–6.01 pmol/L, 9.01–19.5 pmol/L, 0.35–4.94 IU/mL, 0–5.61 IU/mL, and 0–4.11 IU/mL, respectively.

### 2.5 Model establishment

We employed eight machine learning algorithms—logistic regression (LR), support vector machine (SVM), K-nearest neighbors (KNN), adaptive boosting (AdaBoost), multilayer perceptron (MLP), random forest (RF), gradient boosting (GB), and XGBoost (XGB).The data were randomly split into a training set (70%) and a test set (30%) to ensure the adequacy of the training process and the independence of the evaluation process. Missing data is handled using multiple interpolation methods (based solely on the distribution of the training set).To identify factors influencing the progression of coronary heart disease, we first performed a preliminary screening based on correlations and removed redundant features to address multicollinearity issues. Second, the Lasso method was employed to enable the model to automatically learn and select the most valuable subset of features for CHD severity, which were then utilized for subsequent model construction. Subsequently, hyperparameter tuning was performed to improve the performance of these algorithms; this process involved adjusting the parameters using five-fold cross-validation on the training set. Importantly, during the hyperparameter tuning phase, the proposed models relied solely on the training set to obtain the optimal hyperparameters, ensuring that no information from the test set was utilized. Model performance was evaluated on the test set using metrics such as the area under the receiver operating characteristic curve (AUROC), accuracy, precision, recall, and F1 score. In addition, this study employed the Bootstrap resampling method (1,000 iterations) to conduct a statistical comparison of the AUC differences among various machine learning models, in order to verify the significant advantages of ensemble models over traditional linear models. The SHAP method was employed to interpret the models, including calculating feature importance scores and generating SHAP summary plots and dependency plots. These visualizations revealed the specific directional influence of each feature on the model’s output and captured threshold effects.

### 2.6 Statistical analyses

Data analysis was performed using SPSS software, version 26.0, and Python software (version 3.9). Multiple interpolation methods are used to handle missing data. The Kolmogorov–Smirnov test was used to assess the normality of continuous variables. Normally distributed continuous variables are presented as the means ± standard deviations, with intergroup comparisons conducted using the independent samples t test. Skewed continuous variables are presented as medians (interquartile ranges), with intergroup comparisons performed using the Mann–Whitney U test. Categorical variables are expressed as percentages, with intergroup comparisons performed using the chi-square test. Correlation analysis employed Spearman’s correlation coefficient. Multivariate logistic regression analysis was used to examine the relationship between thyroid autoantibodies and the severity of coronary heart disease. P < 0.05 was considered to indicate statistical significance.

## 3 Results

**Figure 1:**
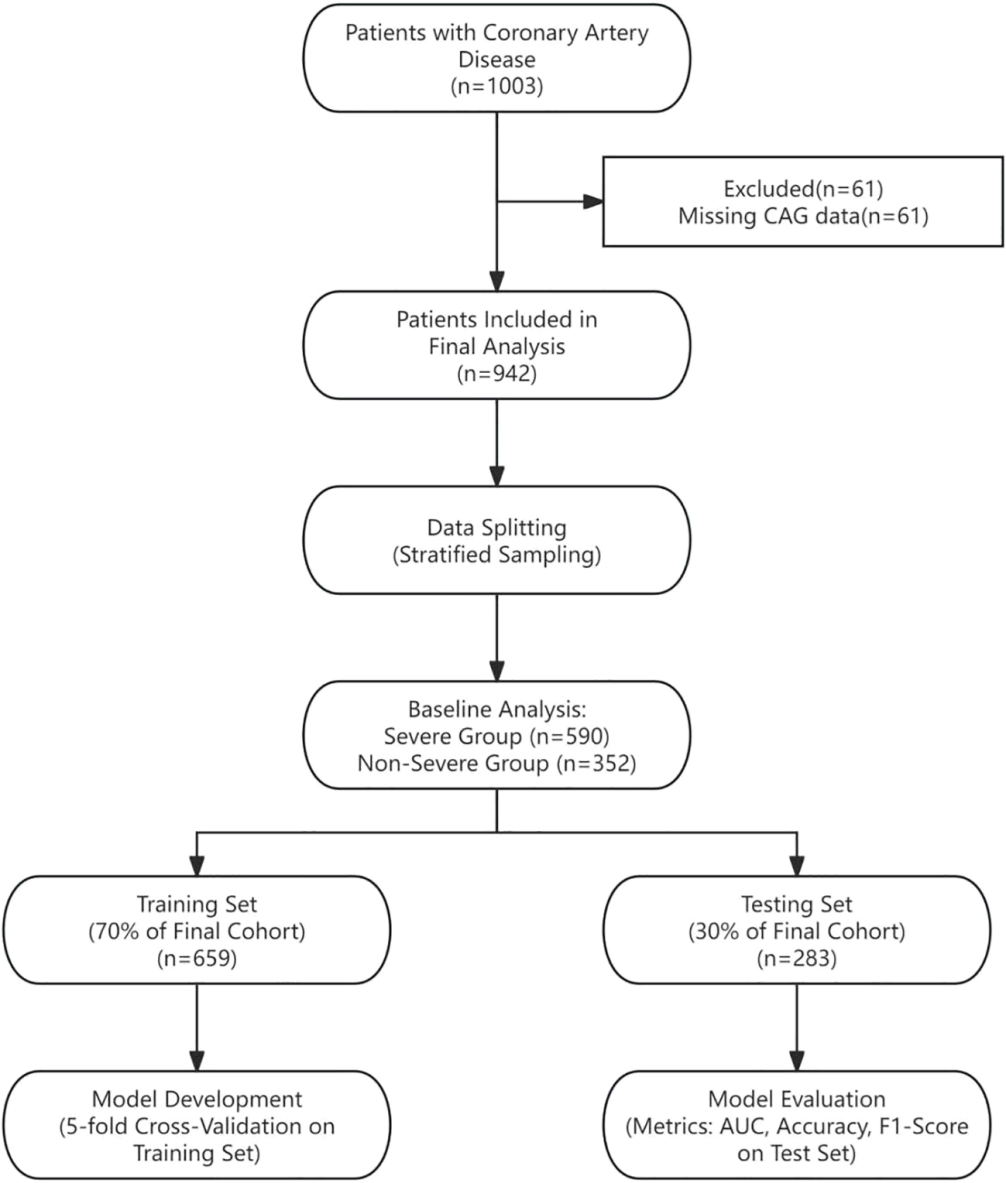
Flow chart of data extraction.

This study included a total of 942 patients. On the basis of the coronary angiography results, all the patients were divided into two groups: the non-severe lesion group (n=352) and the severe lesion group (n=590). The patient screening process is detailed in **Figure 1**.

As shown in **Table 1**, compared with patients in the non-severe lesion group, those in the severe lesion group were older (63.77 ± 10.25 years vs. 62.03 ± 9.52 years, P = 0.008) and had a significantly greater proportion of males (67.6% vs. 56.0%, P < 0.001). In terms of comorbidities, the severe lesion group had significantly greater prevalences of hypertension (54.7% vs. 47.2%, P = 0.029) and diabetes (15.9% vs. 7.4%, P<0.001). Additionally, this group had significantly higher rates of smoking history (24.2% vs. 16.8%, P = 0.009) and alcohol consumption history (13.6% vs. 7.7%, P = 0.008). Among laboratory indicators, glycated hemoglobin (HbA1c), reflecting long-term glycemic control, was significantly elevated in the severe lesion group (median 6.22% vs. 5.94%, P<0.001). Similarly, the levels of B-type natriuretic peptide (BNP), which reflects cardiac workload and function, were significantly greater in the severe lesion group (median 102.05 vs. 66.00 pg/mL, P<0.001). Thyroid hormone levels (FT3, FT4, and TSH) did not significantly differ between the groups (P>0.05). However, the median TPO-Ab concentration was significantly greater in the severe lesion group than in the non-severe lesion group (16.00 IU/mL vs. 2.25 IU/mL, P<0.001), whereas the TgAb concentration was lower in the severe lesion group (1.54 IU/mL vs. 2.79 IU/mL, P = 0.008).

**Table 1:** Demographic information and baseline characteristics of all groups.

Characteristic	Nonsevere (n=352)	Severe (n=590)	P value
Age	62.03 ± 9.52	63.77 ± 10.25	0.008
Sex			<0.001
Female	154(43.8%)	191(32.4%)	
Male	197(56.0%)	399(67.6%)	
BMI	25.06(23.02-27.45)	25.15(23.33-27.16)	0.756
SBP	134.60 ± 16.37	134.61 ± 19.18	0.995
DBP	82.50(77.00-90.00)	82.00(76.00-90.00)	0.358
HbA1c	5.94(5.60-6.60)	6.22(5.77-7.06)	<0.001
Glu	5.47(4.86-6.67)	5.54(4.92-7.08)	0.163
TC	3.95(3.28-4.69)	3.77(3.17-4.56)	0.036
TG	1.50(1.08-2.25)	1.48(1.09-2.02)	0.887
LDL-C	2.35(1.80-3.06)	2.43(1.83-3.15)	0.146
HDL-C	1.08(0.89-1.28)	1.04(0.89-1.20)	0.044
BNP	66.00(30.75-136.50)	102.05(49.63-256.62)	<0.001
Smoking History	59(16.8%)	143(24.2%)	0.009
Alcohol Consumption	27(7.7%)	80(13.6%)	0.008
Hypertension	166(47.2%)	323(54.7%)	0.029
Diabetes	26(7.4%)	94(15.9%)	<0.001
LVEF	63.00(61.00-64.98)	62.70(61.00-64.00)	0.024
LVH	20(5.7%)	34(5.8%)	1.000
Diastolic Dysfunction	66(18.8%)	141(23.9%)	0.078
FT3	3.82(3.49-4.19)	3.80(3.42-4.21)	0.703
FT4	13.00(12.00-14.12)	13.02(11.79-14.11)	0.489
TSH	1.70(1.13-2.67)	1.72(1.08-2.64)	0.478
FT3/FT4	0.29(0.26-0.33)	0.30(0.26-0.34)	0.518
Anti-TPO	2.25(0.77-40.50)	16.00(6.83-29.25)	<0.001
Anti-Tg	2.79(0.89-30.57)	1.54(0.76-20.33)	0.008

**Figure 2:**
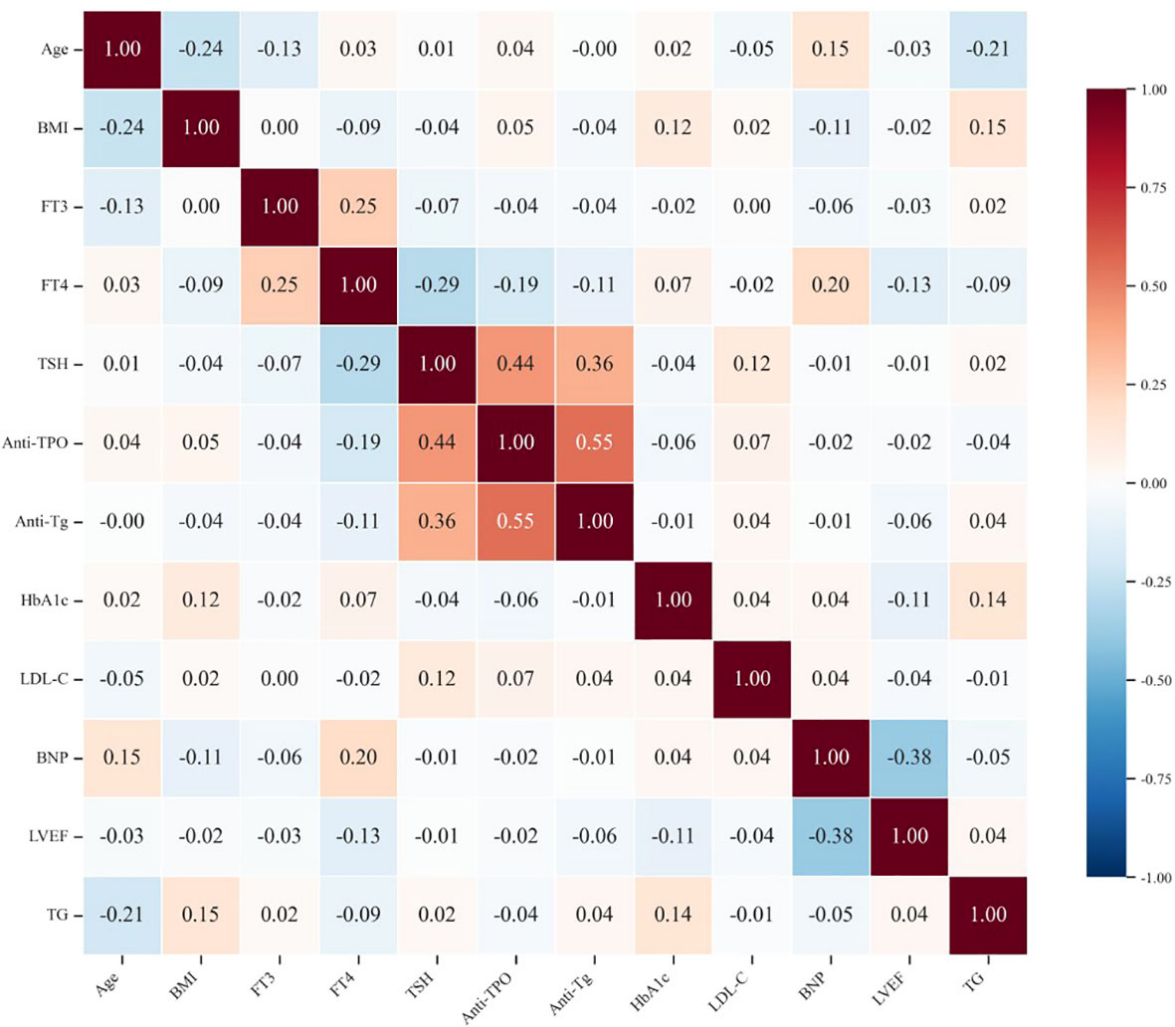
Multivariable correlation heatmap.

To investigate correlations among clinical indicators, we constructed a correlation heatmap (**Figure 2**). The results revealed significant, positive correlations between TPO-Ab and Tg-Ab (r=0.55), with both exhibiting moderate, positive correlations with TSH (TPO-Ab r=0.44; Tg-Ab r=0.36). This finding aligns with the pathophysiological characteristics of autoimmune thyroid disease. A key finding was the extremely weak correlation between thyroid autoantibodies and traditional metabolic risk factors (e.g., LDL-C, HbA1c, and TG), with all correlation coefficients (r) <0.15. These findings suggest that thyroid autoimmunity may influence the progression of coronary atherosclerosis through mechanisms independent of traditional metabolic pathways.

**Figure 3:**
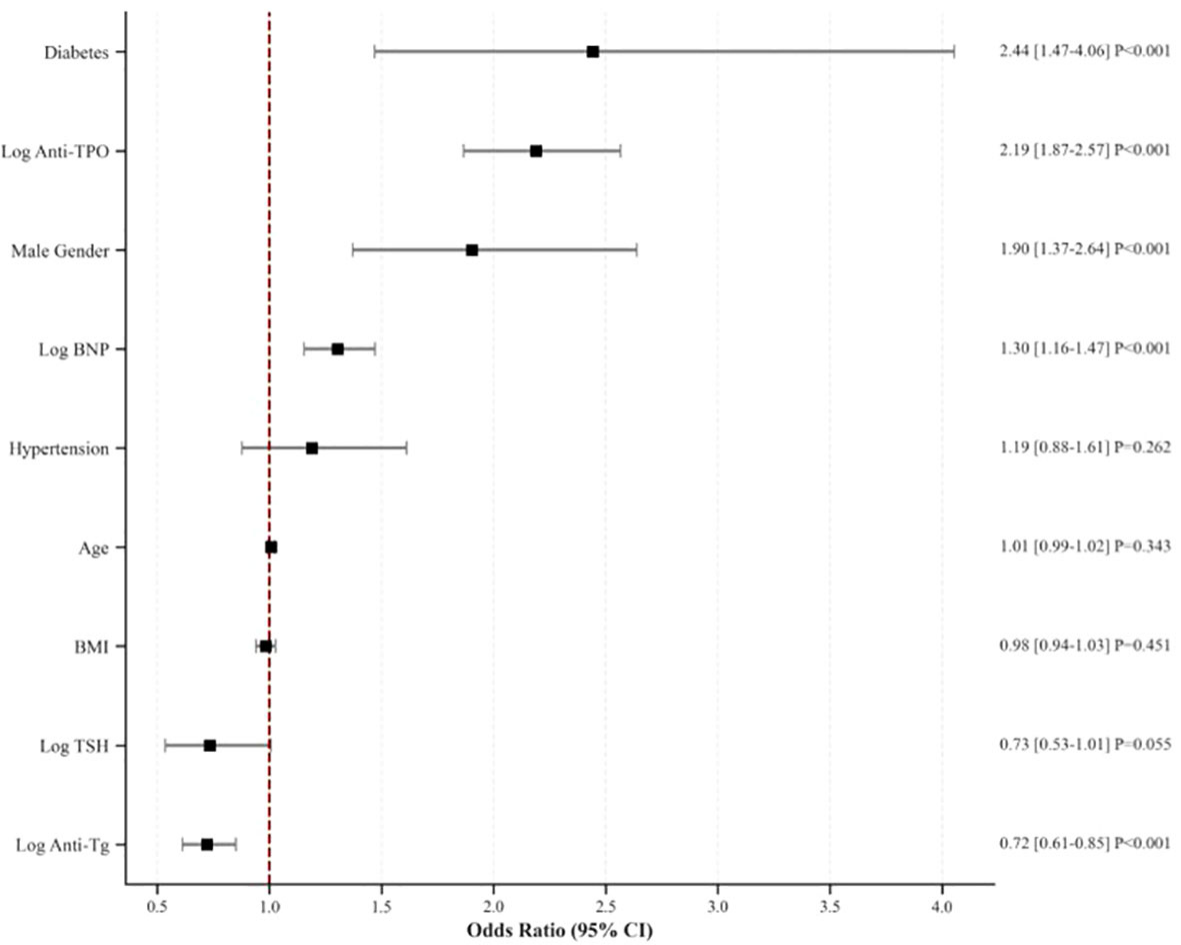
Multivariable forest plot.

To assess the independent predictive value of each indicator for severe coronary artery disease, we constructed a multivariate logistic regression model and included skewed variables after log transformation. As shown in **Figure 3**, after adjusting for confounding factors, such as age, sex, and BMI, log anti-TPO (OR = 2.19; 95% CI: 1.87–2.57; P<0.001), diabetes (OR = 2.44; P<0.001), male sex (OR = 1.90; P<0.001), and log-transformed brain natriuretic peptide (Log BNP; OR = 1.30; P<0.001) emerged as independent risk factors for severe coronary artery disease. Conversely, log Anti-Tg levels were significantly inversely correlated (OR = 0.72, 95% CI: 0.61–0.85; P<0.001). However, given the significant positive correlation between Anti-Tg and Anti-TPO in this cohort (r = 0.55), when two highly correlated variables are included together in a regression model—and one of them (Anti-TPO) is a strong positive predictor of the outcome—the other variable often exhibits a mathematically inverse association after adjustment. This phenomenon strongly suggests a statistical artifact resulting from multicollinearity, rather than a genuine biological “protective” effect.

**Figure 4:**
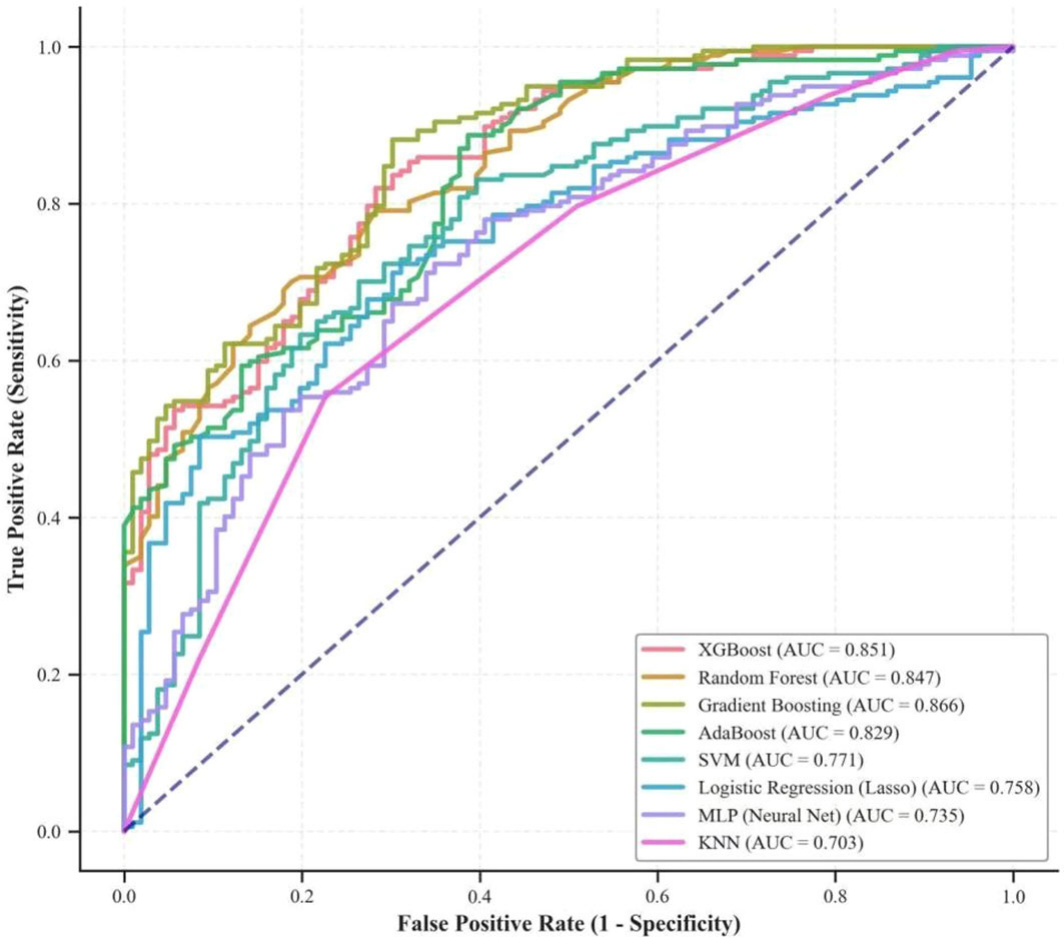
ROC curve comparison of machine learning models.

To explore complex nonlinear relationships among variables and enhance predictive accuracy, we evaluated eight machine learning models, as shown in **Figure 4**. The results indicated that the gradient boosting (GB) model performed best (AUC = 0.855, 95%CI:0.809-0.893), followed by XGBoost (AUC = 0.843, 95% CI: 0.796-0.883) and random forest (RF) (AUC = 0.842, 95% CI: 0.796-0.883). Statistical comparisons using the bootstrap method indicate that there is no statistically significant difference in performance between the best model, Gradient Boosting, and XGBoost (*P* = 0.074) or RF (*P* = 0.342). Compared with traditional models, tree-based ensemble models demonstrate significant advantages. Taking the Gradient Boosting(GB) model as an example, its predictive performance is significantly superior to that of the traditional Lasso model (AUC = 0.758, *P* = 0.002), SVM (AUC = 0.755, *P* < 0.001), and KNN (AUC = 0.722, *P* < 0.001). These results strongly demonstrate,compared to traditional linear models, tree-based ensemble algorithms can more effectively learn from high-dimensional, nonlinear clinical data, thereby more accurately identifying patients with severe coronary artery lesions. **Table 2** details the comprehensive performance metrics for each model. In terms of comprehensive evaluation metrics, tree-based ensemble models also performed exceptionally well. For example, Gradient Boosting(GB) and XGBoost achieved precision scores of 0.809 and 0.798, respectively, and F1 scores of 0.846 and 0.822, respectively. The RF model achieved a recall score of 0.898. These findings suggest that we can select different models depending on the specific context. Based on the results of the performance evaluations and statistical tests described above, and considering that there is no significant difference in predictive performance between the XGBoost algorithm and GB, XGBoost demonstrates exceptional algorithmic efficiency in handling complex feature interactions and exhibits excellent underlying compatibility with the most cutting-edge model interpretability frameworks. Therefore, in the subsequent model interpretability analysis of this study, XGBoost was selected as a representative high-performance “black-box” model to further dissect the intrinsic predictive mechanisms of various clinical features for severe coronary artery disease.

**Table 2:** Performance comparison of machine learning models.

Model	Accuracy	Precision	Recall	F1-Score	AUC (95% CI)	P-value
Gradient Boosting	0.799	0.809	0.887	0.846	0.855 (0.809-0.893)	Reference
XGBoost	0.770	0.798	0.847	0.822	0.843 (0.796-0.883)	0.074
Random Forest	0.777	0.779	0.898	0.835	0.842 (0.796-0.883)	0.342
AdaBoost	0.784	0.776	0.921	0.842	0.829 (0.783-0.871)	0.022
Logistic Regression	0.696	0.805	0.678	0.736	0.758 (0.702-0.812)	0.002
SVM	0.686	0.782	0.689	0.733	0.755 (0.690-0.812)	<0.001
KNN	0.693	0.75	0.763	0.756	0.722 (0.656-0.777)	<0.001
MLP (Neural Net)	0.668	0.732	0.74	0.736	0.708 (0.639-0.768)	<0.001

**Figure 5:**
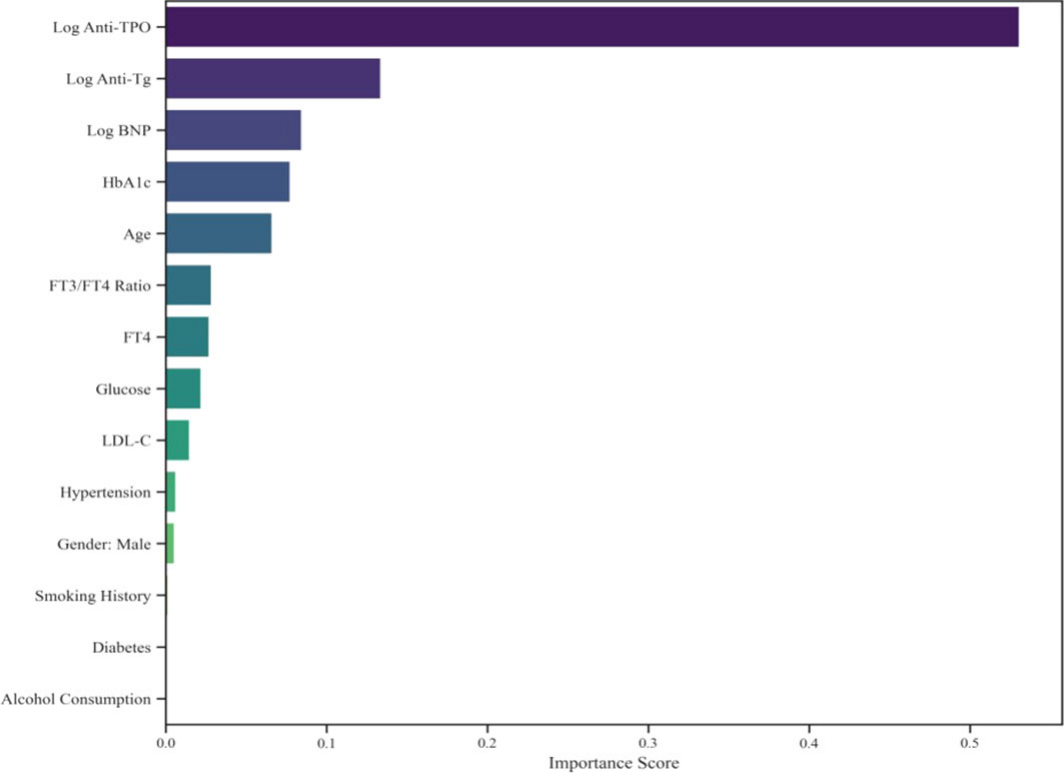
XGBoost feature importance (Top 15).

**Figure 6:**
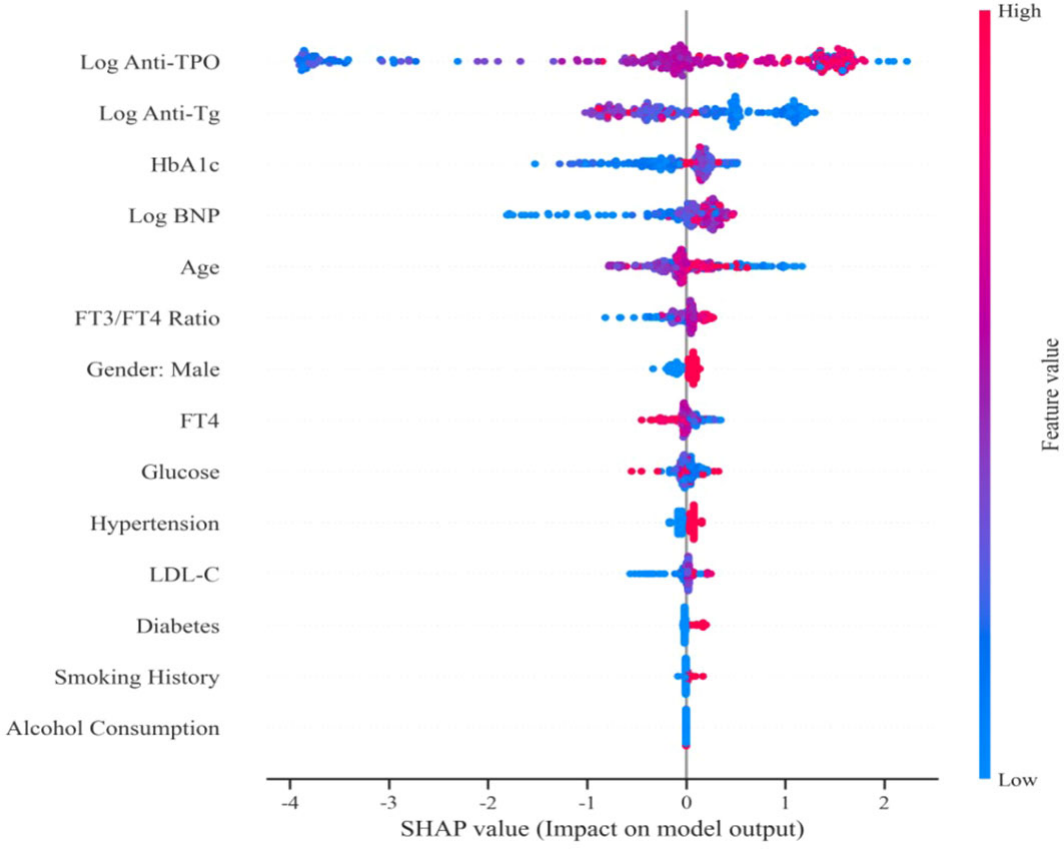
SHAP summary plot.

To assess each indicator’s contribution to model predictions, we applied SHAP values for model interpretation. As shown in **Figure 5**, features were ranked by their importance in predicting coronary heart disease severity, thereby quantifying their overall contributions to model predictions. The results indicate that log anti-TPO is the most critical indicator for predicting severe coronary heart disease, followed by log anti-Tg and log-BNP and then the long-term glycemic control indicators HbA1c and age. The direction and magnitude of each feature’s contribution to the model’s risk prediction are shown in **Figure 6**: SHAP values > 0 indicate a positive influence, whereas SHAP values < 0 indicate a negative influence. Red dots represent higher values, and blue dots represent lower values. The results show that log anti-TPO, log-BNP, HbA1c, and age negatively influence the severity of coronary heart disease, In contrast, Log Anti-Tg tends to be distributed on the negative side of the SHAP values. As discussed in the multivariate regression analysis, this phenomenon is most likely due to a competition for feature weights within the model resulting from multicollinearity between Log Anti-Tg and Log Anti-TPO; therefore, it should not be directly interpreted as indicating an independent cardiovascular protective effect.

**Figure 7:**
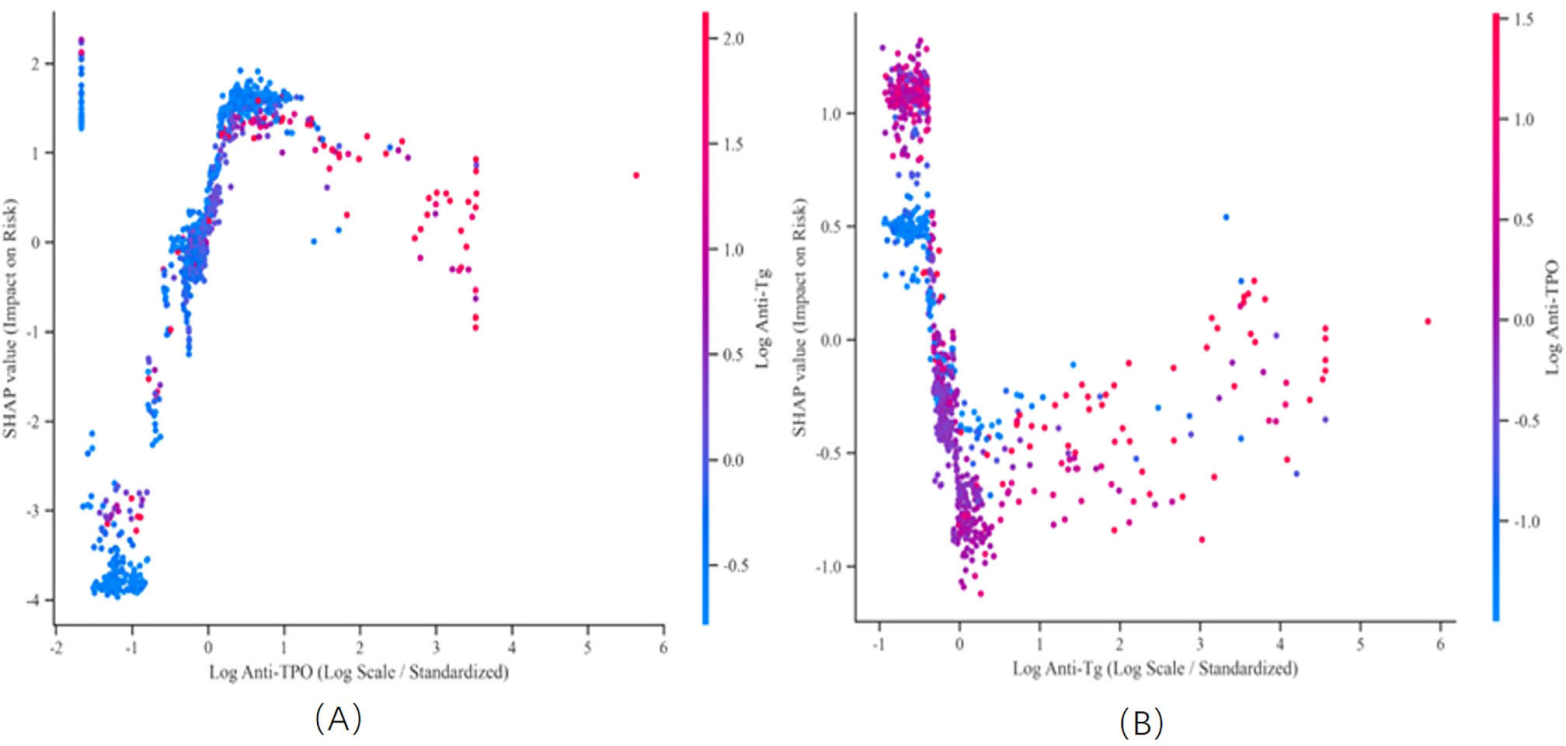
SHAP dependence plot. **(A)** Scatter plot of SHAP impact versus log Anti‑TPO, where points are colored by log Anti‑Tg values (blue to red). **(B)** Scatter plot of SHAP impact versus log Anti‑Tg, where points are colored by log Anti‑TPO values (blue to red).

To investigate the nonlinear threshold effect between variables, we constructed SHAP dependency plots. As shown in **Figure 7A** (log anti-TPO), when log anti-TPO is at low levels, its impact on the risk of severe coronary heart disease is negligible. Once the threshold exceeds zero, its SHAP value sharply increases, causing a steep increase in CHD risk, which then stabilizes at high values. These findings suggest that the pathogenic effect of log anti-TPO may only be activated and rapidly amplified after a specific concentration threshold is exceeded. The data in **Figure 7B** further confirm the negative correlation between log anti-Tg and CHD severity: as log anti-Tg increases, the overall SHAP value decreases. The colors of the points in the figure (driven by Log Anti-TPO) also suggest a complex interaction between the two.

**Figure 8:**
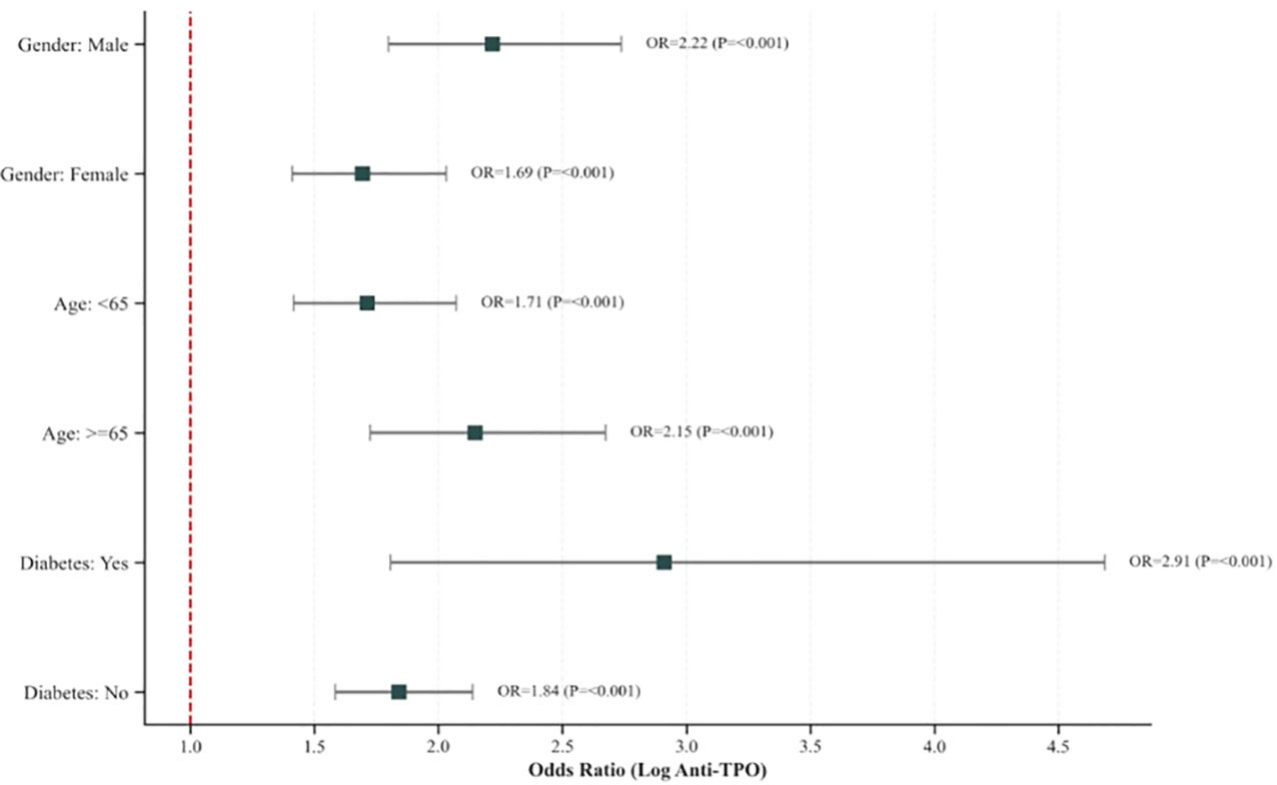
Association between log anti-TPO and risk across subgroups.

To validate the robustness of the predictive value of anti-TPO antibodies, we conducted subgroup analyses. As shown in **Figure 8**, we evaluated the predictive efficacy of anti-TPO levels across different populations. The results demonstrated that log anti-TPO significantly predicted severe coronary heart disease in all the subgroups (P < 0.001), confirming the robustness of its predictive value.

**Figure 9:**
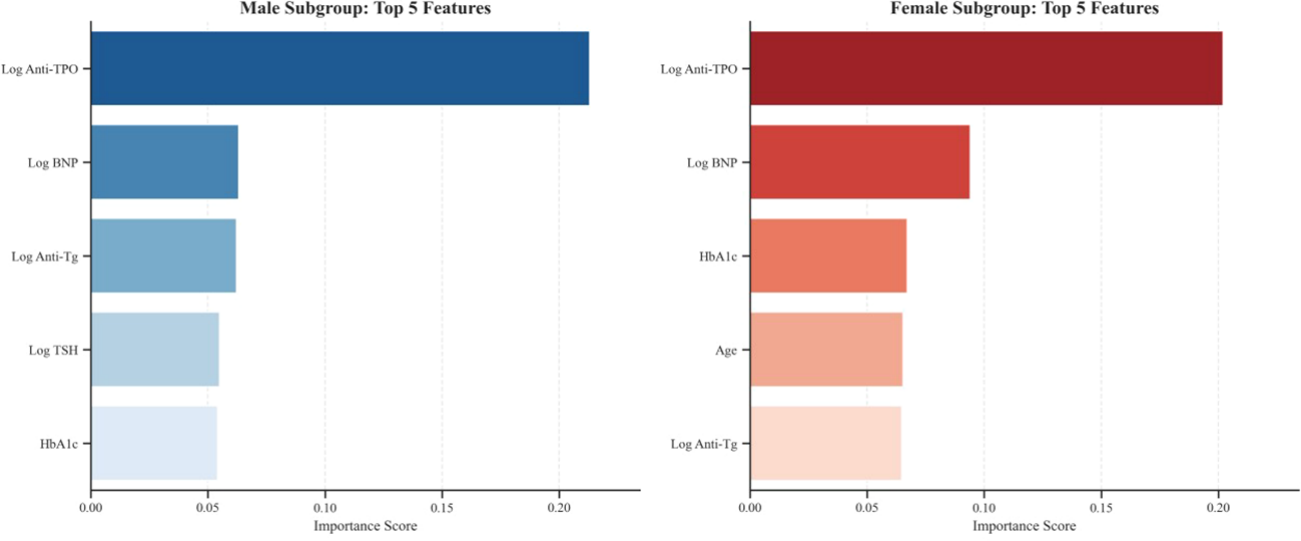
Comparison of feature importance by sex.

Finally, we further compared feature importance by sex, as shown in **Figure 9**. In both the male and female subgroups, log anti-TPO emerged as the most important predictive feature. Additionally, the model revealed subtle differences between sexes: in the male cohort, Log BNP ranked second in importance, behind only log Anti-TPO, whereas in the female cohort, Log BNP and HbA1c held comparable importance, jointly forming the second tier.

## 4 Discussion

This study revealed a positive correlation between TPO-Ab and TgAb levels. Both antibodies were extremely weakly correlated with traditional metabolic risk factors (TG, LDL-C, and HbA1c levels). These findings are consistent with the literature (11). However, more intriguingly, in the multivariate logistic regression model, after adjusting for confounding factors, TPO-Ab is positively correlated with the severity of coronary heart disease, whereas TgAb is negatively correlated with the severity of coronary heart disease. Additionally, through machine learning, TPOAb levels emerged as the most critical predictive factor for severe coronary heart disease. This finding requires interpretation in light of the study population’s characteristics: most patients included in this research had already undergone standardized secondary prevention interventions. These interventions effectively reduced blood pressure and lipid and glucose levels, potentially diminishing the statistical significance of associations between traditional risk factors and disease progression. However, TPO-Ab is not currently a routine indicator in CHD management, and its levels may not be affected by therapeutic interventions. Therefore, this marker may more fully reflect its biological role in determining the severity of CHD. Consequently, the prominent predictive value of TPOAb for CHD severity in this study does not imply that its biological effect surpasses that of traditional factors. Rather, it likely represents residual risk mediated by TPOAb in the context of well-managed traditional risk factors.

For the first time, in individuals with normal thyroid function, we applied a machine learning model system to elucidate the predictive value of thyroid autoantibodies for determining coronary heart disease severity. This approach overcomes the limitations of traditional logistic regression in handling nonlinear relationships and interactions. The objectives of our study differ from those of previous studies (15, 16, 21). Our study focused on the severity of coronary heart disease as the target outcome and revealed a positive correlation between TPO-Ab levels and coronary heart disease severity in patients with normal thyroid function. These findings suggest that TPO-Ab is likely a dynamic marker of coronary heart disease progression. Inflammation is a known cause of endothelial dysfunction, which is associated with atherosclerosis. As a marker of autoimmune thyroiditis, TPO-Ab may trigger autoimmune-mediated inflammatory responses, potentially influencing the progression of atherosclerosis (29). Additionally, some studies have failed to identify an association between TPO-Ab levels and the progression of coronary artery disease (13, 29). For example, a cohort study demonstrated that, after patients who received statin therapy or those with overt or subclinical thyroid disease were excluded, there was no correlation between TPO-Ab levels and the progression of coronary artery calcification (CAC) (29). The aforementioned study examined the relationship between TPO-Ab levels and CAC progression in the ideal setting of untreated individuals without thyroid disease. However, in real-world clinical practice, the vast majority of coronary heart disease patients are receiving treatment for traditional risk factors. Therefore, this study innovatively shifted its focus to residual risk after conventional risk factor management. Even in this context, a positive correlation between TPO-Ab levels and CHD severity was still observed. These findings suggest that TPO-Ab may influence CHD progression through biological pathways independent of traditional risk factors. Naturally, this conclusion requires further validation in prospective cohort studies, and its biological mechanisms warrant further elucidation. Additionally, our study revealed that TgAb was negatively correlated with CHD severity. Previous studies has shown that TgAb positivity is an independent protective factor against coronary heart disease. Additionally, subjects with TgAb exhibit milder coronary artery stenosis (13). Another study indicated that TgAb exerts a protective effect against metabolic disorders in individuals with normal thyroid function. This protective mechanism is hypothesized to be associated with carbonic anhydrase (CA) activity, as reduced CA activity may increase TgAb production through elevated iodine uptake (30). However, most of the studies cited above regarding the protective effects of TgAb do not report the degree of colinearity between the two antibodies, and the robustness of their results warrants further evaluation. In this study, TgAb showed a moderate positive correlation with TPO-Ab (r = 0.55), and TPO-Ab was a strong risk factor for the severity of coronary heart disease (OR = 2.19). When these two highly correlated variables were included simultaneously in the regression model, the coefficient for TgAb was “pushed” in the opposite direction, a classic statistical artifact resulting from multicollinearity. Therefore, the negative correlation observed for TgAb in this study is more likely to reflect a mathematical bias in the effect estimate rather than an independent protective mechanism. Future studies should measure both antibodies simultaneously and report their correlation, using appropriate methods to account for multicollinearity, in order to reliably determine whether TgAb possesses independent clinical value.

Our subgroup analysis revealed that TPO-Ab was more strongly associated with severe coronary heart disease in males, individuals aged ≥65 years, and those with diabetes. The sex-specific differences in the association strength between TPO-Ab and coronary heart disease severity may reflect complex interactions among sex hormones, immune regulation, and cardiac metabolic risk characteristics (29). In females, estrogen suppresses vascular calcification and delays the progression of coronary heart disease by regulating a series of molecular and cellular events, including hypoxia-inducible factor-1α signaling, autophagy, and estrogen receptor α-dependent transactivation of growth-inhibitory specific genes (31). Furthermore, animal studies have demonstrated that estrogen therapy can reduce the progression of experimentally induced atherosclerosis. Aging is a major risk factor for the high incidence of chronic diseases, such as metabolic disorders and cardiovascular diseases. With advancing age, immune system function gradually declines, placing the body in a state of chronic inflammation (32). The combined action of circulating inflammatory mediators and TPO antibodies may directly damage vascular endothelial cells, leading to atherosclerosis. This process could explain why severe coronary heart disease is more prevalent in the elderly population. The risk of autoimmune-mediated coronary heart disease progression is independent of traditional cardiovascular risk factors, although it can be exacerbated by them (11). Thyroid autoimmunity and diabetes may exhibit synergistic pathogenic effects, thereby exacerbating the severity of coronary atherosclerosis. The immune system plays a role in immune surveillance, but excessive nutritional exposure can trigger inflammation and autoimmune diseases, leading to metabolic reprogramming of immune cells (33, 34). Autoimmune disorders frequently cause cellular dysfunction, which in turn leads to impaired glucose metabolism (35). Poor glycemic control may exacerbate immune-mediated vascular injury through elevated glycated hemoglobin levels, inducing oxidative stress and endothelial dysfunction. This effect synergizes with TPO-Ab-mediated inflammation to accelerate lipid deposition and vascular remodeling (36). These abnormalities may in turn trigger or exacerbate autoimmune dysfunction, creating a vicious cycle (37).

This study has several limitations. First, This study employed a cross-sectional design, and all participants had been diagnosed with coronary artery disease (CAD). Another possible explanation is that severe atherosclerosis and its associated systemic inflammatory response lead to elevated TPO-Ab titers, thereby making TPO-Ab a concomitant biomarker reflecting disease severity. Therefore, prospective cohort studies are needed in the future to investigate this causal relationship. Second, this study was designed as a single-center retrospective study, which inevitably involves selection bias and lacks an external validation cohort. Although we employed rigorous cross-validation to prevent overfitting, The generalizability of this model to other independent cohorts needs to be validated in future multicenter studies. Finally, residual confounding risks remain in this study, particularly due to the failure to adjust for the use of key medications such as statins. Given the immunomodulatory properties of statins, their exclusion as covariates may introduce some confounding in the association between antibody levels and the severity of coronary artery disease.

## 5 Conclusion

In summary, this study employed traditional statistical methods and eight machine learning approaches to evaluate, for the first time, the predictive value of thyroid autoantibodies for determining coronary heart disease severity in individuals with normal thyroid function. Among the machine learning models, the gradient-boosted tree model demonstrated the best performance. These findings provide new insights into the role of thyroid autoantibodies in CHD progression.

## Data Availability

The raw data supporting the conclusions of this article will be made available by the authors, without undue reservation.

## References

[1] Virani SS Alonso A Aparicio HJ Benjamin EJ Bittencourt MS Callaway CW . Heart disease and stroke statistics-2021 update: A report from the American Heart Association. Circulation. (2021) 143:e254–743. doi: 10.1161/cir.0000000000000485. PMID: 33501848 PMC13036842

[2] Center For Cardiovascular Diseases The Writing Committee Of The Report On Cardiovascular H Diseases In China N . Report on cardiovascular health and diseases in China 2023: An updated summary. Biomed Environ Sci: BES. (2024) 37:949–92. 10.3967/bes2024.162 39401992

[3] Xu C Wu M Zhang X Shen K Guo Y Yuan J . Carotid intima thickness and elasticity combined with MHR predicting the severity of coronary artery stenosis in patients with premature coronary artery disease. BMC Cardiovasc Disord. (2025) 25:241. doi: 10.1186/s12872-025-04693-w. PMID: 40169942 PMC11963636

[4] Si J Chen L Yu C Guo Y Sun D Pang Y . Healthy lifestyle, DNA methylation age acceleration, and incident risk of coronary heart disease. Clin Epigenet. (2023) 15:52. doi: 10.1186/s13148-023-01464-2. PMID: 36978155 PMC10045869

[5] Roth GA Abate D Abate KH Abay SM Abbafati C Abbasi N . Global, regional, and national age-sex-specific mortality for 282 causes of death in 195 countries and territories, 1980-2017: A systematic analysis for the Global Burden of Disease Study 2017. Lancet (London England). (2018) 392:1736–88. doi: 10.1016/s0140-6736(18)32203-7. PMID: 30496103 PMC6227606

[6] Su J Li Z Huang M Wang Y Yang T Ma M . Triglyceride glucose index for the detection of the severity of coronary artery disease in different glucose metabolic states in patients with coronary heart disease: A RCSCD-TCM study in China. Cardiovasc Diabetol. (2022) 21:96. doi: 10.21203/rs.3.rs-1537070/v1. PMID: 35668496 PMC9169264

[7] Fröhlich E Wahl R . Thyroid autoimmunity: Role of anti-thyroid antibodies in thyroid and extra-thyroidal diseases. Front Immunol. (2017) 8:521. doi: 10.3389/fimmu.2017.00521 28536577 PMC5422478

[8] Zhang J Gao Y Li Y Teng D Xue Y Yan L . The presence of serum TgAb suggests lower risks for glucose and lipid metabolic disorders in euthyroid general population from a national survey. Front Endocrinol. (2020) 11:139. doi: 10.3389/fendo.2020.00139. PMID: 32256451 PMC7093715

[9] Carbotta G Tartaglia F Giuliani A Carbotta S Tromba L Jacomelli I . Cardiovascular risk in chronic autoimmune thyroiditis and subclinical hypothyroidism patients. A cluster analysis. Int J Cardiol. (2017) 230:115–9. doi: 10.1016/j.ijcard.2016.12.066. PMID: 28038798

[10] Dogdus M Diker S Yenercag M Gurgun C . Evaluation of left atrial and ventricular myocardial functions by three-dimensional speckle tracking echocardiography in patients with euthyroid Hashimoto’s thyroiditis. Int J Cardiovasc Imaging. (2021) 37:459–65. doi: 10.1007/s10554-020-02011-3. PMID: 32897525

[11] Porsch F Binder CJ . Autoimmune diseases and atherosclerotic cardiovascular disease. Nat Rev Cardiol. (2024) 21:780–807. doi: 10.1038/s41569-024-01045-7. PMID: 38937626

[12] Szyper-Kravitz M Marai I Shoenfeld Y . Coexistence of thyroid autoimmunity with other autoimmune diseases: Friend or foe? Additional aspects on the mosaic of autoimmunity. Autoimmunity. (2005) 38:247–55. doi: 10.1080/08916930500050194. PMID: 16126513

[13] Yang L Zhang M Zhang H Zheng G Xu C Li G . Association of thyroid autoimmunity with the presence and severity of coronary atherosclerosis in patients undergoing coronary angiography. Medicine. (2022) 101:e30881. doi: 10.1097/md.0000000000030881. PMID: 36181027 PMC9524898

[14] Asvold BO Bjøro T Platou C Vatten LJ . Thyroid function and the risk of coronary heart disease: 12-year follow-up of the HUNT study in Norway. Clin Endocrinol. (2012) 77:911–7. doi: 10.1111/j.1365-2265.2012.04477.x 22724581

[15] Collet TH Bauer DC Cappola AR Asvold BO Weiler S Vittinghoff E . Thyroid antibody status, subclinical hypothyroidism, and the risk of coronary heart disease: An individual participant data analysis. J Clin Endocrinol Metab. (2014) 99:3353–62. doi: 10.1210/jc.2014-1250. PMID: 24915118 PMC4154087

[16] LeGrys VA Funk MJ Lorenz CE Giri A Jackson RD Manson JE . Subclinical hypothyroidism and risk for incident myocardial infarction among postmenopausal women. J Clin Endocrinol Metab. (2013) 98:2308–17. doi: 10.1210/jc.2012-4065. PMID: 23539723 PMC3667262

[17] Hak AE Pols HA Visser TJ Drexhage HA Hofman A Witteman JC . Subclinical hypothyroidism is an independent risk factor for atherosclerosis and myocardial infarction in elderly women: The Rotterdam Study. Ann Internal Med. (2000) 132:270–8. doi: 10.7326/0003-4819-132-4-200002150-00004. PMID: 10681281

[18] Wells BJ Hueston WJ . Are thyroid peroxidase antibodies associated with cardiovascular disease risk in patients with subclinical hypothyroidism? Clin Endocrinol. (2005) 62:580–4. doi: 10.1111/j.1365-2265.2005.02262.x. PMID: 15853828

[19] Song JL Hu JW Li LR Xu ZL Li JJ Sun SR . Association of thyroid autoimmunity with extra-thyroid diseases and the risk of mortality among adults: Evidence from the NHANES. Front Endocrinol. (2024) 15:1323994. doi: 10.3389/fendo.2024.1323994. PMID: 38405150 PMC10884096

[20] Hysaj O Efthimiou O Collet TH Cappola AR Alwan H Gussekloo J . Thyroid antibody status, thyroid function, and the risk of coronary heart disease and stroke: An individual participant data analysis from 14 cohorts. Eur J Endocrinol. (2025) -:-–-. doi: 10.1093/ejendo/lvaf209. PMID: 41137464 PMC13200528

[21] Shimizu Y Kawashiri SY Noguchi Y Nagata Y Maeda T Hayashida N . Normal range of anti-thyroid peroxidase antibody (TPO-Ab) and atherosclerosis among eu-thyroid population: A cross-sectional study. Medicine. (2020) 99:e22214. doi: 10.1097/md.0000000000022214. PMID: 32957357 PMC7505314

[22] Liu X Wu S Yang Y Li Y Zhang X Liu R . Development of machine learning predictive model for type 2 diabetic retinopathy using the triglyceride-glucose index explained by SHAP method. Front Endocrinol. (2025) 16:1631647. doi: 10.3389/fendo.2025.1631647. PMID: 41293734 PMC12640817

[23] Nemati S Holder A Razmi F Stanley MD Clifford GD Buchman TG . An interpretable machine learning model for accurate prediction of sepsis in the ICU. Crit Care Med. (2018) 46:547–53. doi: 10.1097/ccm.0000000000002936. PMID: 29286945 PMC5851825

[24] Bhardwaj V Sharma A Parambath SV Gul I Zhang X Lobie PE . Machine learning for endometrial cancer prediction and prognostication. Front Oncol. (2022) 12:852746. doi: 10.3389/fonc.2022.852746. PMID: 35965548 PMC9365068

[25] Shi Y Ma L Chen X Li W Feng Y Zhang Y . Prediction model of obstructive sleep apnea-related hypertension: Machine learning-based development and interpretation study. Front Cardiovasc Med. (2022) 9:1042996. doi: 10.3389/fcvm.2022.1042996. PMID: 36545020 PMC9760810

[26] Lawton JS Tamis-Holland JE Bangalore S Bates ER Beckie TM Bischoff JM . 2021 ACC/AHA/SCAI guideline for coronary artery revascularization: A report of the American College of Cardiology/American Heart Association Joint Committee on Clinical Practice Guidelines. Circulation. (2022) 145:e18–e114. doi: 10.1161/cir.0000000000001038. PMID: 34882435

[27] Sacks DB Arnold M Bakris GL Bruns DE Horvath AR Lernmark Å . Guidelines and recommendations for laboratory analysis in the diagnosis and management of diabetes mellitus. Diabetes Care. (2023) 46:e151–e99. doi: 10.1093/clinchem/48.3.436 PMC10516260 37471273

[28] Du S Neiman A Batis C Wang H Zhang B Zhang J . Understanding the patterns and trends of sodium intake, potassium intake, and sodium to potassium ratio and their effect on hypertension in China. Am J Clin Nutr. (2014) 99:334–43. doi: 10.3945/ajcn.113.059121. PMID: 24257724 PMC3893725

[29] Meneghini V Tebar WR Generoso G Janovsky CC Lotufo PA Bittencourt MS . Thyroid peroxidase antibodies and coronary artery calcification: Results from the ELSA-Brasil cohort study. J Endocrinol Invest. (2025) -:-–-. doi: 10.1007/s40618-025-02751-w. PMID: 41251950

[30] Duntas LH . The catalytic role of iodine excess in loss of homeostasis in autoimmune thyroiditis. Curr Opin Endocrinol Diabetes Obes. (2018) 25:347–52. doi: 10.1097/med.0000000000000425. PMID: 30124478

[31] Woodward HJ Zhu D Hadoke PWF MacRae VE . Regulatory role of sex hormones in cardiovascular calcification. Int J Mol Sci. (2021) 22. doi: 10.3390/ijms22094620. PMID: 33924852 PMC8125640

[32] Barbé-Tuana F Funchal G Schmitz CRR Maurmann RM Bauer ME . The interplay between immunosenescence and age-related diseases. Semin Immunopathol. (2020) 42:545–57. doi: 10.1007/s00281-020-00806-z. PMID: 32747977 PMC7398288

[33] Pearce EL Pearce EJ . Metabolic pathways in immune cell activation and quiescence. Immunity. (2013) 38:633–43. doi: 10.1016/j.immuni.2013.04.005. PMID: 23601682 PMC3654249

[34] Scheja L Heeren J . Introduction to the special issue on dietary control of immunometabolism. Semin Immunopathol. (2018) 40:141–4. doi: 10.1007/s00281-017-0667-4. PMID: 29222582

[35] Wu Y Shi X Tang X Li Y Tong N Wang G . The correlation between metabolic disorders and Tpoab/Tgab: A cross-sectional population-based study. Endocrine Pract. (2020) 26:869–82. doi: 10.4158/ep-2020-0008. PMID: 33471678

[36] Wu Y Sun J Ni Y Wang M Qiao H . Hashimoto’s thyroiditis and thyroid hormone sensitivity in euthyroid individuals: Their association with carotid plaque in northeast China. Front Endocrinol. (2025) 16:1605875. doi: 10.3389/fendo.2025.1605875. PMID: 41040864 PMC12483847

[37] Wang R Green DR . Metabolic reprogramming and metabolic dependency in T cells. Immunol Rev. (2012) 249:14–26. doi: 10.1111/j.1600-065x.2012.01155.x. PMID: 22889212 PMC3422760

